# PPARs and Angiogenesis—Implications in Pathology

**DOI:** 10.3390/ijms21165723

**Published:** 2020-08-10

**Authors:** Nicole Wagner, Kay-Dietrich Wagner

**Affiliations:** Université Côte d’Azur, CNRS, INSERM, iBV, 06107 Nice, France; kwagner@unice.fr

**Keywords:** peroxisome proliferator-activated receptor, angiogenesis, endothelial cells, signaling pathways, cancer, cardiovascular disease, retinal angiogenesis, endometrium, placenta development

## Abstract

Peroxisome proliferator-activated receptors (PPARs) belong to the family of ligand-activated nuclear receptors. The PPAR family consists of three subtypes encoded by three separate genes: PPARα (NR1C1), PPARβ/δ (NR1C2), and PPARγ (NR1C3). PPARs are critical regulators of metabolism and exhibit tissue and cell type-specific expression patterns and functions. Specific PPAR ligands have been proposed as potential therapies for a variety of diseases such as metabolic syndrome, cancer, neurogenerative disorders, diabetes, cardiovascular diseases, endometriosis, and retinopathies. In this review, we focus on the knowledge of PPAR function in angiogenesis, a complex process that plays important roles in numerous pathological conditions for which therapeutic use of PPAR modulation has been suggested.

## 1. Introduction

Peroxisome proliferator-activated receptors (PPARs) belong to the nuclear hormone receptor family. Three PPAR isotypes with different tissue distribution, ligand specificity, and metabolic regulatory activities exist: PPARα, PPARβ/δ, and PPARγ. PPARs regulate many metabolic pathways upon activation by endogenous ligands, such as fatty acids (FAs) and derivatives, or synthetic modulators, which bind to the ligand-binding domain of the receptor, triggering a conformational change. Subsequent recruitment of coactivators to the PPAR/retinoid X receptor heterodimer assembled at specific DNA response elements, named PPAR responsive elements (PPREs), results in transactivation or repression of target genes (reviewed in [1]).

PPAR expression in endothelial cells has been reported two decades ago [2] and all PPARs are modulating angiogenesis. PPARα and PPARγ were identified to mediate anti-angiogenic processes, in contrast, PPARβ/δ emerged as a pro-angiogenic nuclear receptor (reviewed in [3]). However, today, there exist controversial results if PPAR ligands act as inhibitors or inducers of angiogenesis. The intention of this review is to assess the data regarding the effects of PPAR modulation in distinct in vitro and specific pathophysiological and therapeutic settings with the aim to provide practical considerations for their use in different diseases implicating angiogenesis or vascular abnormalities. The multiple functions of PPARs in modulation of metabolic disorders e.g., diabetes mellitus, obesity, or non-alcoholic fatty liver disease are reviewed in detail elsewhere [1,4,5,6,7,8] and are beyond the scope of this review.

## 2. PPARs and Cellular Models of Angiogenesis

In 1999, two independent papers showed that PPARs are expressed in endothelial cells (HUVEC model) and treatment with PPARγ agonists inhibits proliferation, tube formation, [9] and migration [10] and induces apoptosis [2]. These principle observations were confirmed nearly twenty years later using a more sophisticated Tie2CrePPARγ(flox/flox) mouse model and isolation of pulmonary microvascular endothelial cells [11]. Different PPARγ agonists inhibited VEGF-and FGF-induced angiogenesis in vitro [12,13,14]. These antiangiogenic effects of PPARγ activation were attributed to increased endothelial nitric-oxide (NO) production and subsequent maxi potassium channel opening [15]. Alternatively, PPARγ agonists might inhibit angiogenesis by suppressing PKCα -and CREB-mediated COX-2 expression in human endothelial cells [16] and activate p38 MAPK pathway and inhibit phosphorylation of p42/44 MAPK, while VEGFs and FGFs only stimulate p38MAPK, but do not affect p42/44 phosphorylation [17]. Importantly, treatment with PPARγ agonists also reduced expression of VEGF receptors in HUVECs [9,18] although later the opposite result was published in the same HUVEC model [19]. PPARγ agonists inhibited VEGF promoter activity and expression in endometrial cells [20]. In bovine retinal endothelial cells, 15d-PGF2 and pioglitazone suppressed VEGFR2 expression and promoter activity due to interference with SP1 and SP3 binding [21]. In contrast, PPARγ activation increased VEGF production in smooth muscle and macrophage cell lines [22,23], which might not be pro-angiogenic as VEGF receptors are downregulated in endothelial cells in response to PPARγ stimulation. In cardiac myofibroblasts, PPARγ activation induced VEGF and VEGF receptor expression [24]. Also in a bladder cancer cell line, VEGF expression was upregulated in response to stimulation with PPARα, PPARβ/δ, and PPARγ agonists, while another bladder cancer cell line responded only to PPARβ/δ activation [25]. PPARβ/δ activation stimulated VEGF expression in breast and prostate cancer and HUVEC cells and increased endothelial cell proliferation [26]. The in vivo relevance of this observation remained unclear. Piqueras et al. showed that PPARβ/δ activation induces VEGF expression, endothelial cell proliferation, aortic sprouting, and in vivo angiogenesis in Matrigel plug assays [27]. More recent results show some different metabolic alterations of HUVEC cells in response to VEGF or PPARβ/δ activation using GW0742 [28] and our group confirmed increased endothelial cell proliferation of HUVEC cells as well as upregulation of pro-angiogenic gene expression upon PPARβ/δ activation with GW0742 [29].

Also the angiogenic capacity of endothelial progenitor cells depends on PPARβ/δ, but also on COX-1 and PGI(2) synthase [30]. PPARβ/δ agonist treatment induced heme oxygenase-1 expression, which renders endothelial cells in culture resistant to cellular stress induced by hydrogen peroxide or leptin [31]. PGC-1α represents an important co-regulator of PPARβ/δ for transcriptional activation of heme oxygenase-1 [31]. In contrast to these findings, treatment with very high concentrations of PPARβ/δ agonists inhibited VEGFR2 expression and in vitro angiogenesis of HUVECs [32]. Furthermore, the PPARβ/δ ligand L-165041 was shown to inhibit VEGF-stimulated angiogenesis by suppressing the cell cycle progression independently of PPARβ/δ [33].

Ibuprofen, an activator of PPARα [34], was found to increase expression of CD36, the anti-angiogenic Thrombospondin-1 (TSP-1) receptor [35] in human melanoma cells [36]. In line with an anti-angiogenic action of PPARα, it has been reported that Gypenoside XLIX, a naturally occurring gynosaponin and natural PPARα agonist, as well as Wy14643, a synthetic PPARα agonist, inhibited tissue factor (TF) promoter activity in human monocytes, which could be blocked by the selective PPARα antagonist MK-886 [37]. TF expression in cancers contributes to a shift in the balance between endogenous pro-angiogenic and anti-angiogenic factors thereby facilitating tumor progression [38]. Anti-angiogenic properties of pomegranate peel extract have been suggested to be mediated by PPARα and PPARγ, as simultaneous treatment of HUVEC cells with pomegranate extract and antagonists of PPARα and PPARγ inhibited the anti-angiogenic response of endothelial cells to pomegranate extract [39]. The PPARα agonist fenofibrate inhibited endothelial cell proliferation, migration, tube formation, and induced apoptosis at high concentrations, which was attributed to decreased Akt activation and Cox-2 expression [40]. Targeting vascular NADPH oxidase 1 resulted in upregulation of PPARα and subsequent inhibition of endothelial cell migration and tube formation, which is in agreement with an anti-angiogenic role of PPARα [41]. The experimental PPARα agonist (R)-K-13675 reduced expression of inflammatory markers, but did not affect proliferation or tube formation of human coronary endothelial cells [42]. Whether these effects are specific for the agonist or the cell line remained unclear. Although all the experiments using cellular model systems described in this chapter contribute to the understanding of the molecular actions of PPARs in angiogenic modulation, it remains unclear to what extent they describe the in vivo function of PPARs in influencing angiogenesis, as the concentrations of compounds used largely differ and the PPAR expression and activation level in each cell type might be different.

## 3. PPARs and Tumor Angiogenesis

### 3.1. PPARα

In non-melanoma skin cancer, PPARα expression was less likely to occur in actinic lesions and squamous cell carcinoma than in normal skin, however, no correlation with microvessel densities could be established. In contrast, PPARβ/δ was upregulated in malignant lesions and the observed PPARβ/δ overexpression correlated with higher vessel densities in these tumor samples [43]. In contrast to these findings in human tumors, an elegant in vivo study by Kaipainen and colleagues demonstrated that PPARα favors tumor progression in mice. Using subcutaneous implantation of syngenic Lewis lung carcinoma (LLC1) or B16 melanoma cells in PPARα deficient mice, the authors demonstrated that tumor growth is prevented in the absence of PPARα. PPARα deficiency induced a strong inflammatory response with an excess of TSP-1 production leading to suppression of tumor angiogenesis. Inhibition of TSP-1 restored tumor growth and bone marrow transplantation and granulocyte depletion experiments showed that PPARα expressing granulocytes, probably myeloid derived suppressor cells, are critically involved in tumor angiogenesis and growth [44]. However, the same group demonstrated one year later that treatment with the PPARα agonist fenofibrate inhibited tumor growth in syngenic LLC1 or B16 melanoma tumor bearing mice, due to an increase of TSP-1 and inhibition of tumor angiogenesis. The authors contribute this paradox of PPARα effect to a bi-phasic dose-response curve of host tissue: Both, very high, or, in contrast, very low concentrations of PPARα result in suppression of tumor angiogenesis [45]. In favor of an anti-tumorigenic PPARα action, Yokoyama and colleagues reported that treatment with clofibric acid, a PPARα agonist, reduced ovarian cancer cell growth and tumor angiogenesis in xenotransplanted animals. This was accompanied by a significantly reduced expression of the pro-angiogenic factors prostaglandin E (2) (PGE(2)) and vascular endothelial growth factor (VEGF) [46]. PPARα anti-angio-and tumorigenic action was further supported by a study from Pozzi and coworkers. They demonstrated that PPARα agonists downregulate the biosynthesis of epoxyeicosatrienoic acids, expoxygenase products, which are pro-angiogenic. Using a PPARα-humanized mouse (hPPARα) model, where the human PPARα gene was introduced onto a PPARα deficient background, they determined that tumor angiogenesis and growth were inhibited in animals which received Wyeth (pirinixic acid, a potent PPARα agonist). They further demonstrated a reduced endothelium-associated epoxygenase expression in the PPARα agonist administered animals, concluding that the tumor-angiogenesis inhibition by PPARα activation involves reduced vascular expoxygenase expression [47]. In line with this, PPARα agonists reduced expression of epoxyeicosatrienoic acids, leading to decreased vascularization and growth of non-small cell lung cancers in murine K-Ras or orthotopic models [48]. However, a recent study by Wu and colleagues showed that the PPARα agonist AVE8134 indeed reduced epoxyeicosatrienoic acid fabrication, but increased their hydroxyl product, 11-hydroxyeicosatetraenoic acids (11-HETE). 11-HETE in contrast promote angiogenesis and tumor growth, therefore neutralizing the beneficial effect of the PPARα agonist AVE8134. Addition of the cyclooxygenase (COX) inhibitor Indomethacin neutralized the undesired effects of AVE8134 and restored the beneficial anti-tumor properties of PPARα agonism [49]. One mechanism favoring tumor angiogenesis is the inhibition of PPARα by the NADPH oxidase NOX1. NOX1 deficient mouse liver endothelial cells displayed 5-fold higher PPARα levels than their wildtype derived counterparts and impaired angiogenic properties, which could be rescued by administration of the PPARα antagonist GW6471. Inducing syngenic LLC1 or B16 tumors, the authors showed that NOX1 deficient mice displayed less tumor vascularization and growth. To further consolidate the relevance of PPARα repression by NOX1 for tumor angiogenesis, the authors induced syngenic tumors in PPARα deficient and wildtype mice and treated them with the NOX1 inhibitor GKT136901. Tumor vascularization was inhibited in wildtype, but not in PPARα knockout animals, which confirmed the repression of PPARα through NOX1 as an important event in favor of enhanced tumor angiogenesis [41]. PPARα and PPARγ have also been suggested as mediators of the anti-angio- and tumorigenic effects of pomegranate extract as their respective antagonists abolished the beneficial effects of pomegranate extract on cancer vascularization and growth [39,50]. In contrast to these studies, Huang and colleagues did not observe a reduction of tumor vascularization upon PPARα activation with fenofibrate in mice bearing B cell lymphomas [51].

### 3.2. PPARβ/δ

The peroxisome-proliferator activated receptor PPARβ/δ favors tumor angiogenesis. As already mentioned, PPARβ/δ was found to be overexpressed in malignant squamous cell carcinoma and its expression correlated with higher vessel densities [43]. A supreme study confirmed the significance of PPARβ/δ for tumor angiogenesis. Using cDNA arrays of human microvascular cells submitted to pro-angiogenic stimuli, PPARβ/δ was identified to be the hubnode of the “angiogenic switch” in cancers, marking the shift in the angiogenic balance to a pro-angiogenic state of the tumor, favoring progression and metastasis. Correlation analysis of different human cancer types further confirmed the link between high PPARβ/δ expression and advanced stage of tumor progression and metastasis. These findings were confirmed in vivo using PPARβ/δ knockout mice bearing syngenic subcutaneously implanted LLC1 lung or B16 melanoma tumors. Cancer growth and angiogenesis were found to be dramatically reduced in PPARβ/δ deficient animals [52]. Müller-Brüsselbach and colleagues further demonstrated a significantly diminished tumor blood flow due to a hyperplastic non-functional microvasculature in LLC1 and B16 tumors of PPARβ/δ knockout mice, leading to the impairment of tumor growth. PPARβ/δ had therefore been suggested to be a pre-requisite for microvessel maturation and differentiation [53]. In human colorectal cancer samples, Yoshinaga and colleagues correlated PPARβ/δ and cyclooxygenase 2 (COX-2) expression with VEGF expression, microvascular densities, and incidence of venous vessel invasion. Their results suggested that simultaneous expression of PPARβ/δ and COX-2 increased angiogenesis and metastasis in colon cancer, thereby worsening the patients prognosis [54]. Recently, it has been demonstrated that high expression levels of PPARβ/δ in cancer cells significantly contribute to tumor angiogenesis. Lung metastasis formation by tail vein injection of different cancer cell types (B16 melanoma, LLC1 lung carcinoma, HCT116 colon carcinoma, Panc-02 pancreatic carcinoma, and 4T1 breast cancer) was diminished upon knockdown of PPARβ/δ in the cancer cells. The fewer lung metastases formed by the cancer cells with knockdown of PPARβ/δ displayed significantly reduced microvessel densities. Angiogenic VEGF and Interleukin 8 expression levels were dramatically reduced in cancer cells silenced for PPARβ/δ. These findings clearly indicate that PPARβ/δ is pro-angio-tumorigenic independent of its source of expression: Normal host cells which contribute to the tumor stroma or cancer cells [55]. Our group showed that treatment of LLC1 tumor bearing mice with the PPARβ/δ agonist GW0742 increased tumor angiogenesis and growth. We then addressed the question if solely selective overexpression of PPARβ/δ in endothelial cells would be sufficient to enhance tumor angiogenesis and growth independently of the status of PPARβ/δ expression in cancer and non-endothelial host cells. To investigate this, we made use of mice with inducible vascular specific overexpression of PPARβ/δ [56] with subcutaneously implanted LLC1 tumors. We observed increased tumor angiogenesis, growth, and metastasis formation upon vessel specific overexpression of PPARβ/δ. RNA sequencing of tumor sorted endothelial cells revealed a high number of upregulated pro-angiogenic genes in response to PPARβ/δ increase. By combining top ten network analysis with a search for PPAR responsive elements, we identified the platelet-derived growth factor (PDGF)/platelet-derived growth factor receptor (PDGFR) pathway, tyrosinkinase KIT (c-Kit), and the VEGF/vascular endothelial growth factor receptor (VEGFR) pathway as mediators of the pro-angiogenic tumor promoting effect of PPARβ/δ [29].

### 3.3. PPARγ

In contrast to PPARβ/δ, most studies identified PPARγ as an inhibitor of tumor angiogenesis. Although no upregulation or correlation with vascular density could be detected in skin squamous cell carcinoma [43], PPARγ was less expressed in high grade and more vascularized gliomas than in low grade gliomas which display less microvascular density. PPARγ expression in gliomas further positively correlated with anti-angiogenic TSP-1 expression [57]. Panigrahy and colleagues demonstrated PPARγ expression in tumor endothelium, reduced tumor growth and metastatic spreading of subcutaneously implanted LLC1, glioblastoma, liposarcoma, and rhabdomyosarcoma upon treatment with the PPARγ agonist rosiglitazone. Double-labeling of vessels for PPARγ and proliferating nuclear antigen (PCNA) revealed significantly reduced endothelial cell proliferation in the tumor specimens from animals which received rosiglitazone [58]. It has been further shown that PPARγ ligands increase the success of tumor anti-angiogenic therapies with exogenous TSP-1 or its peptide derivative ABT510. The PPARγ agonists 15d-PGJ2, rosiglitazone, and troglitazone increased TSP-1 receptor CD36 expression on the endothelial cell surface, thereby improving the sensitivity to exogenous TSP-1 or its peptide derivative ABT510 and inhibiting angiogenesis through induction of endothelial cell apoptosis. Simultaneous PPARγ activation improved the anti-tumor activity of ABT510 in bladder carcinoma bearing mice [59]. As already mentioned, PPARγ antagonists have been demonstrated to abolish the beneficial effects of pomegranate extract in inhibition of tumor angiogenesis and growth [39,50]. PPARγ has also been shown to inhibit tumor angiogenesis and growth of A594 lung cancers in vivo by blocking the production of CXCL1, CXCL5, and interleukin 8, or CXCL8 (CXC chemokines with a specific amino acid sequence of glutamic acid-leucine-arginine (ELR) before the first cysteine of the CXC motif (ELR-positive)). This is mediated through transcriptional inhibition of nuclear factor kappa-light-chain-enhancer of activated B cells (NF-κB) activity, a transcription factor which regulates expression of chemokines [60]. The PPARγ agonist RS5444 inhibited tumor vascularization and growth of xenotransplanted anaplastic thyroid carcinomas. Anti-tumor activity could be further enhanced by combination therapy with paclitaxel chemotherapy [61]. Inhibition of tumor angiogenesis and growth in vivo by PPARγ activation with thiazolidinediones has further been reported for ovarian carcinoma [62,63], and pancreatic cancer [64]. Combination of radiotherapy with the PPARγ agonist rosiglitazone enhanced the effectiveness of radiotherapy against tumor angiogenesis, distant metastasis formation, and tumor recurrence in animal models with subcutaneous breast or colon cancer cell implantation [65]. Berger and colleagues identified activation of suppressor of cytokine signaling 3 (SOCS3) by PPARγ agonists as another pathway implicated in suppression of tumor angiogenesis and growth. Activation of SOCS3 by PPARγ inhibited differentiation of proinflammatory T helper (T_h_) 17 cells and their secretion of Interleukin (IL)-17. PPARγ activation with n-3 fatty acid docosahexaenoic acid (DHA) in vivo inhibited tumor vascularization and progression in a IL-17 dependent manner, but failed to reduce tumor vessel formation and growth in immunodeficient or IL-17 knockout animals, suggesting that the tumor angiogenesis inhibiting effects of PPARγ activation depend on T cells and the secretion of the pro-inflammatory cytokine IL-17 [66]. Deletion of PPARγ in the mammary epithelium promoted mammary stem cell (MSCs) expansion favoring angiogenesis and breast cancer growth. PPARγ deficient breast cancers were insensitive to chemotherapy, but normalization of the abundant tumor vasculature with the anti-angiogenic drug sunitinib increased efficiency of cytostatic chemotherapy. The PPARγ agonist rosiglitazone increased micro RNA miR-15a expression which inhibited angiopoietin-1, resulting in decreased angiogenesis, MSC expansion, and tumor growth in vivo [67]. In contrast to these studies, Tian and coworkers demonstrated that activated PPARγ promoted tumor angiogenesis and growth in breast cancer. Ligand activation of PPARγ induces a conformational change in the receptor, which the authors mimicked by mutation before introducing it in NAFA cells derived from oncogenic MMTV-ErbB2 mice. They compared the constitutively active PPARγ (PγCA) mutant with the wild-type PPARγ in ErbB2-induced mammary tumorigenesis by implantation into immunocompetent FVB mice. Enhanced tumor growth associated with increased angiogenesis and higher numbers of endothelial stem cells was observed in animals implanted with PγCA cells. Genome-wide expression profiling identified a group of genes within the angiogenesis pathway, including angiopoietin-like 4 (Angptl4), fibroblast growth factor 1 (Fgf1), and pleiotrophin (Ptn) as targets of activated PPARγ favoring tumor angiogenesis [68]. An important study from the group of Michalik further cautions the risks of the use of thiazolidinedione PPARγ agonists in cancer patients. They could not demonstrate any correlation of PPARγ expression with the different stages of melanoma disease, but evidenced an increased release of pro-tumorigenic cytokines (Interleukin 1β (IL1 β, Interleukin 6 (IL6)), chemokines (granulocyte-macrophage colony-stimulating factor (GM-CSF)), and angiogenic factors (angiopoietin-like 4 (ANGPTL4), Interleukin 8 (IL8)) by melanoma cells treated with the thiazolidinedione rosiglitazone. The pro-tumorigenic secretome of rosiglitazone treated melanoma cells activated nonmalignant stromal cells, fibroblasts, immune, and endothelial cells to promote tumor growth in vivo. PPARγ activation with thiazolidinediones could therefore have deleterious effects in patients with cancer [69]. In conclusion, PPARβ/δ clearly favors tumor angiogenesis (reviewed in [70]). Although PPARα and PPARγ have initially been described as anti-angiogenic (reviewed in [3]), conflicting results have been obtained over the time. The different approaches described in this chapter are summarized in a simplified manner in Table 1. In vitro versus in vivo studies, the use of immunodeficient mice displaying only a partial real response to in vivo tumor growth, different behavior of divergent cancer cell types, differential dose-response kinetics, and cross-activation of different PPARs upon ligand incitement contribute to the plethora of reasons for these conflicting results. Unfortunately, in none of the clinical studies concerning the use of PPAR ligands in cancer (listed in chapter seven), the effects on tumor vascularization have been investigated. Ultimately, therapeutic modulation of any PPAR should be considered with great care given its potential activation of tumor angiogenesis.

## 4. PPARs and Cardiovascular Disease

Endothelial dysfunction is a typical feature of type 2 diabetes. Endothelial progenitor cells (EPC) contribute to angiogenesis and endothelial function. Pistroch et al. showed that the migratory function of EPCs is reduced in diabetic patients compared to controls and rosiglitazone treatment normalized the migratory function and increased the number of EPCs [71]. In diabetic mice, blood flow recovery was impaired after hindlimb ischemia compared to non-diabetic controls. Treatment with PPARγ agonists partially restored blood flow recovery and increased the capillary density in ischemic hindlimbs of control and diabetic mice [72] and in muscles of diabetic rats [73]. These positive effects of PPARγ agonist treatment were NO-dependent and related to eNOS upregulation [74]. Also peroxisome-proliferator-activated receptor-gamma coactivator-1alpha (PGC-1α) contributes to recovery from hindlimb ischemia via direct induction of VEGF expression independent on hypoxia-inducible factor 1 (Hif-1) [75].

Coronary arteriosclerosis with lumen obstruction is frequently treated with angioplasty. After angioplasty, inflammation, adventitial angiogenesis, constrictive remodeling and intimal hyperplasia result in re-stenosis. Kasai et al. demonstrated that treatment with the PPARα agonist fenofibrate reduced inflammation, angiogenesis, and re-stenosis in a porcine angioplasty model [76]. However, the PPARα agonist fenofibrate induced angiogenesis in the rat hindlimb ischemia model and in the myocardium of diabetic rats [77,78]. Whether these differences are related to the investigated species or the doses and time frame remains an open question. Importantly, fenofibrate reduced the risk for first and minor amputations in diabetic patients in the Fenofibrate Intervention and Event Lowering in Diabetes (FIELD) study [79], which was probably through non-lipid mechanisms and might involve induction of angiogenesis. Deng et al. showed that fenofibrate normalizes the function of endothelial progenitor cells in diabetic mice, which led to increased angiogenesis and accelerated wound closure. This was attributed to an increase in NO production and inhibition of the Nod-like receptor protein 3 inflammasome pathway [80].

In rats with focal cerebral ischemia, treatment with the PPARγ agonist rosiglitazone increased angiogenesis, improved functional recovery, reduced apoptosis, and diminished the lesion size [81]. In human aortic segments with early atheromatous lesions, however, endogenous lipid mediators of PPARγ were enriched in the intimal layer, which was associated with enhanced VEGF production of smooth muscle cells in the media layer and subsequent increased angiogenesis. PPARγ antagonists blocked these effects while rosiglitazone mimicked the pro-angiogenic effects [82]. These findings seem to be in contrast to the in vitro angiogenesis data for PPARγ, but are highly relevant as they take into account the interplay between the different human cell types in situ. As angiogenesis favors intraplaque hemorrhage and plaque rupture, a potential use of PPARγ agonists in the setting of atherosclerosis should be considered with care. Small clinical trials showed some benefits of pioglitazone on plaque inflammation [83], and reduction of systolic and diastolic blood pressure, a decrease in the duration and frequency of angina attacks, regression of atherosclerosis of the carotid vessels, and decrease in the thickness of the intima-media complex [84]. A decrease in intima-media complex thickness in response to pioglitazone treatment had been described already earlier [85]. Pioglitazone did not affect endothelin-1 activity as the main endpoint in another clinical trial [86], but reduced aortic stiffness, rheumatoid arthritis disease activity and CRP levels in patients with rheumatoid arthritis [87]. Pioglitazone as well as fenofibrate treatment of obese, glucose tolerant men reduced inflammation, improved markers of endothelial function and reduced arterial stiffness [88]. In larger clinical studies, the dual PPARα/γ agonist aleglitazar (AleCardio and ALEPREVENT randomized clinical trials) showed no significant improvement of cardiovascular disease, but multiple significant side effects [89,90,91,92]. In the PROactive study, pioglitazone reduced cardiovascular complications [93], while independent studies showed that rosiglitazone increased the rate of cardiovascular death [94,95]. These effects are most likely no related to angiogenesis, but due to their differing effects on lipid levels [4,5,96].

Pharmacological PPARβ/δ activation in mice resulted in rapid remodeling of muscles with an increase in oxidative fibers, myonuclear accretion, and angiogenesis [97,98]. Furthermore, exercise-induced angiogenesis in skeletal muscle requires the presence of PGC-1α as co-activator. Mice lacking PGC-1α were not able to respond to exercise with an increase in muscle angiogenesis [99]. Five years later, Han and colleagues claimed that the role of PPARβ/δ activation in vascular biology and skeletal muscle is widely unknown and reported increased angiogenesis and muscle regeneration in a hindlimb ischemia model in mice in response to GW501516 treatment, which was attributed to direct MMP9 activation followed by degradation of insulin-like growth factor-binding protein 3 and resulting IGF-1 receptor activation in surrounding target cells [100]. Also treatment with the PPARβ/δ agonist GW0742 or the pan PPAR agonist bezafibrate or a Chinese traditional medicine compound which activates PPARβ/δ increased capillary density in the hindlimb ischemia model in control and diabetic rats and mice [101,102,103]. In the heart, pharmacological PPARβ/δ activation induced rapid onset angiogenesis and cardiac hypertrophy without functional impairment, which we could attribute to direct transcriptional activation of calcineurin [104]. These modifications resembled exercise-induced phenotypes and thus, were in line with potential therapeutic benefits. However, it remained unclear whether pharmacological PPARβ/δ activation in the heart acts on cardiomyocytes, which secondary leads to increased vascularization or the opposite. Thus, we generated a model with inducible endothelial-specific overexpression of PPARβ/δ. These mice showed increased angiogenesis and cardiac hypertrophy suggesting that cardiomyocyte growth is secondary to the angiogenic process in this model. Surprisingly, functional recovery after experimental myocardial infarction was not improved, but cardiac fibrosis increased [56,105]. A similar observation of increased angiogenesis and fibrosis without functional benefit after infarction in rats treated with a pharmacological PPARβ/δ agonist was published by Park et al. [106]. Hypertrophy was not investigated in this study in detail.

## 5. PPARs and Ocular Angiogenesis

Xin et al. did not only investigate in vitro angiogenesis, but also used a rat corneal angiogenesis assay. They mechanically wounded the cornea and locally applied VEGF, which increased angiogenesis. This response was blunted by co-administration of a PPARγ agonist [9]. This finding was confirmed in choroidal neovascularization models in rats and monkeys [107]. Curiously, a similar result was published later using the PPARγ agonist pioglitazone. VEGF increased ocular angiogenesis. Addition of pioglitazone did not have a significant effect; nevertheless the authors concluded that pioglitazone reduced ocular angiogenesis [108]. Dietary supplementation with ω-3 long-chain polyunsaturated fatty acids reduced lesion sizes in a model of age-related macular degeneration in mice, which was attributed to upregulation and activation of PPARγ and reduced angiogenesis. 17,18-epoxyeicosatetraenoic acid and 19,20-epoxydocosapentaenoic acid were identified as key lipid mediators of disease resolution [109]. These data are in agreement with the studies mentioned above suggesting an antiangiogenic role of PPARγ in ocular disease. Unfortunately, besides these exciting findings in mice, ω-3 long-chain polyunsaturated fatty acid supplementation did not become a cure for age-related macular degeneration in humans. In patients treated with the PPARγ agonist rosiglitazone, the onset of proliferative diabetic retinopathy was delayed and the vision loss reduced compared to the control group, which was attributed to reduced angiogenesis [110]. In line with this, 4-hydroxy-docosahexaenoic acid, a natural 5-lipoxygenase catalyzed product from ω-3 polyunsaturated fatty acids effectively reduced pathological retinal neovascularization in a mouse model of oxygen-induced retinopathy via PPARγ [111,112]. Also 15-lipoxygenase-1 gene transfer decreased pathological retinal angiogenesis in a comparable model via PPARγ and down-regulation of Vegfr-2 [113]. In a mouse model of ischemic retinopathy, animals receiving an intravitreal injection of peroxisome proliferator-activated receptor-γ coactivator-1α (PGC-1α) showed increased VEGF levels in the retina and enhanced angiogenesis [114]. Whether this intravitreal injection of a normally nuclear expressed PPAR co-activator is relevant for retinal pathologies is questionable.

In the murine corneal model of angiogenesis, Pola et al. determined that prostacyclin analogues, which act non-specifically on PPARs, induce angiogenesis via VEGF upregulation while cicaprost, a prostacyclin analogue acting on IP receptors, but not on PPARs failed to induce angiogenesis [115]. Later, the same group confirmed that PPARα (WY14643) or PPARγ (GW1929) activation induces corneal angiogenesis in a VEGF-dependent manner [116]. WY14643 increased IL-1β-induced inflammatory cytokines in primary human corneal epithelial cells, keratocytes, and retinal endothelial cells and upregulated VEGF expression in corneal epithelial cells and keratocytes suggesting a proinflammatory and proangiogenic role of PPARα in ocular cells [117]. However fenofibrate suppressed retinal and choroidal neovascularization via CYP2C inhibition as well as by acting as an agonist of PPARα in vitro as well as in mice in vivo [118]. Very high doses of the pan PPAR agonist bezafibrate inhibited inflammatory responses and VEGF expression in retinal microvascular endothelial cells and human retinal pigment epithelial cells [119]. In the Fenofibrate Intervention and Event Lowering in Diabetes (FIELD) study, the requirement for laser treatment for all retinopathy was significantly lower in the fenofibrate group than in the placebo group independent on plasma lipid levels [120] suggesting a function on angiogenesis.

The PPARβ/δ agonist GW0742 increased retinal endothelial cell tubulogenesis, while the antagonist GSK0660 reduced tube formation in a dose-dependent manner. More importantly, GSK0660 was able to inhibit ocular neoangiogenesis and inflammation in a rat model of hyperoxia/hypoxia [121,122] while GW501516 inhibited re-epithelialization and induced angiogenesis in corneal wounds [123]. GSK0660 blocked the effect of TNFα on the expression of cytokines involved in leukocyte recruitment i.e., CCL8, CCL17, and CXCL10 and thus the authors concluded that it might block TNFα-induced retinal leukostasis [121,124]. Choroidal neovascular lesions were smaller in aged PPARβ/δ knockout compared to wild-type mice and GSK0660 resulted in a significant inhibition of neovascular lesion size, and extracellular matrix deposition in aged mice [125].

## 6. PPARs and Rheumatoid Arthritis

Arthritis is an inflammatory joint disease, which is in the early phase characterized by vascularization and inflammation. Already in the 1990s, it had been reported that PPARγ activation inhibits macrophages/monocytes and the inflammatory cytokine production, which are important for arthritis [126,127]. Consequently, PPARγ activation reduced experimental arthritis [128,129,130,131,132] and cartilage-specific knockout of PPARγ resulted in development of osteoarthritis in mice [133,134]. PPAR activators partially inhibited the expression of vascular cell adhesion molecule-1 (VCAM-1) and monocyte binding to human aortic endothelial cells [135].

A pilot clinical study by Bongartz et al., using pioglitazone showed positive responses in 6 out of 10 patients. As major side effects, edema and weight gain were observed [136]. Given the nature of the study, angiogenesis and inflammation in the joints could not be investigated in detail. In another small clinical trial, pioglitazone only modestly reduced rheumatoid arthritis disease activity [137] and vascular function [138]. Morin, a flavonoid from dietary plants was identified as PPARγ agonist, which attenuates synovial angiogenesis and arthritis via the PPARγ-PTEN-PI3K/Akt pathway [139].

PPARα activation with fenofibrate was first reported in a clinical case study for the treatment of rheumatoid arthritis [140]. In a following experimental study, it was shown that fenofibrate treatment inhibits NF-kappaB activation, cytokine production, and the development of rheumatoid arthritis [141]. Fenofibrate treatment resulted in improvement of osteoarthritis symptoms, reduction in triglyceride levels, decreased circulating IL-10 levels, while circulating endothelial progenitor cell counts were unaffected in another small pilot study [142].

Most of the studies mentioned above focused on the role of PPARs in modulation of inflammation, cartilage, and fibroblast function. Only studies which to some extend paid attention to the angiogenic process or endothelial progenitor cells were described here.

## 7. PPARs and Uterine and Placental Angiogenesis

PPARγ is highly expressed in cytotrophoblasts and syncytiotrophoblasts in human placentas and in the trophoblast zone of rodents [143,144,145]. In addition, PPARα and PPARβ/δ were detected in the syncytiotrophoblast layer of human and rodent placentas [146,147,148].

Knockout of PPARγ in mice results in lethality between day 9.5 and 11.5 of embryonic development due to placental defects [149,150]. To explore whether this placental defect was the only cause of embryonic lethality in PPARγ knockout embryos, Nadra et al., established an epiblastic-specific deletion strategy of PPARγ to demonstrate that the expression of PPARγ in the placenta is sufficient to rescue the embryonic lethality of PPARγ knockout embryos [151,152]. Rosiglitazone treatment in wild-type mice during later stages of embryonic development resulted in a disorganization of the placental layers and an altered placental microvasculature, accompanied by decreased expression of proangiogenic genes such as Prl2c2, vascular endothelial growth factor, and Pecam1 [151], which points to the limitations of the use for PPARγ agonists for the treatment of metabolic syndrome during pregnancy. In line with this, treatment of pregnant rats with the PPARγ antagonist T0070907 induced key features of preeclampsia, including elevated mean arterial blood pressure, proteinuria, endothelial dysfunction, reduced pup weight, and increased platelet aggregation. VEGF levels were reduced and plasma soluble fms-like tyrosine kinase 1 increased in response to the treatment. Placentas of T0070907-treated rats were less differentiated, had increased cellular proliferation, and were strongly positive for CD-31 staining indicating increased angiogenesis [153]. In contrast in pigs, adhesive, proliferative and migratory capabilities of endothelial cells were potentiated by rosiglitazone and suppressed by T0070907 [154].

In a mouse model of endometriosis, Nenicu et al. described that telmisartan, a partial agonist of PPARγ, which additionally blocks angiotensin II type 1 receptors, reduced functional microvessel density and blood perfusion, inhibited immune cell infiltration and cell proliferation which resulted in smaller lesion sizes [155]. How this ectopic tissue transplantation in mice translates to the human situation remained an open question.

Also knockout of PPARβ/δ has been shown to result in placental defects and midgestation lethality in the majority of embryos [156]. PPARβ/δ plays a central role at various stages of pregnancy; while maternal PPARβ/δ is critical to implantation and decidualization, embryonic PPARβ/δ is vital for placentation [157]. Treatment of pregnant rats with the PPARβ/δ agonist GW501516 induced placental malformations [158]. Angiogenesis or vessel density were unfortunately not determined in detail in this model.

## 8. PPAR Modulators in Clinical Studies

Given the interest in therapeutic PPAR modulation it is astonishing to note that only few clinical trials, most of them concerning cancer, are listed in the major clinical trials database (https://clinicaltrials.gov) (Table 2). Only in one trial concerning cardiovascular disease and rheumatoid arthritis, effects of PPAR regulation on angiogenesis were investigated [87]. This is unfortunate in the case of cancer trials, given the tumor-promoting effects of angiogenesis which might even dominate anti-proliferative actions of PPAR modulators on tumor cells [29]. Use of PPAR modulators in the therapy of cardiovascular diseases is likely to influence angiogenesis which in turn affects the outcome of such therapies [159]. As PPAR modulation regulates ocular and uterine angiogenesis, it is of great importance to investigate the effects on angiogenesis in therapeutic interventions using PPAR regulators against diabetic retinopathy or endometriosis, as both pathologies depend on an excessive vascularization [160,161], which is also the case in rheumatoid arthritis [162]. Therefore, taking into account the effects on angiogenesis in clinical studies implying PPAR regulators might help for a better understanding of the clinical outcomes of such trials.

## 9. Conclusions

Several in vitro and animal in vivo studies suggest that mainly PPARγ, but also PPARα has antiangiogenic effects. Agonists for both nuclear receptors are in clinical use. PPARβ/δ in contrast has pro-angiogenic functions. Therefore, a potential pharmacological application might be critical in the settings of cancer. In general, small clinical studies showed some positive outcomes for the above described pathophysiological conditions. Nevertheless, for a clinical benefit, not only endothelial function and angiogenesis have to be considered, but also the interplay between the different cellular systems i.e., vascular, immune and stromal cells.

In general, the concept of the role of angiogenesis for therapeutic interventions should be critical revised as for example we and others showed that PPARβ/δ stimulation induces angiogenesis in the heart, but unexpectedly this had no positive functional effects after myocardial infarction. Similar effects might play a role under several pathophysiological situations, which makes it necessary to investigate not only the angiogenic process, but also functional consequences. The influence of PPAR modulation on angiogenesis remains an extremely interesting topic and should be taken into account seriously when considering PPAR regulators for therapeutic use in pathological conditions strongly depending on angiogenesis. Currently, the role of modulated angiogenesis through therapeutic intervention via PPAR modulation presented in this review is a hypothesis based on mainly experimental studies. Further large-scale clinical trials would be needed to justify this application.

## Figures and Tables

**Table 1:** PPAR Modulation in in vivo Studies of Tumor Angiogenesis.

PPAR	Condition	Final Effect on Tumor Angiogenesis	Reference
PPARα	PPARα knockout mice	↑	[44]
PPARα	PPARα agonist fenofibrate	↓	[45]
PPARα	PPARα agonist clofibric Acid	↓	[46]
PPARα	PPARα agonist Wyeth (pirinixic acid)	↓	[47]
PPARα	PPARα agonists bezafibrate and Wyeth-14,643	↓	[48]
PPARα	PPARα agonist AVE8134	↑↓	[49]
PPARα	PPARα repression by NOX1 inhibitor GKT136901	↓	[41]
PPARα	PPARα antagonist GW6471	↓	[50]
PPARβ/δ	PPARβ/δ knockout mice	↑	[52]
PPARβ/δ	PPARβ/δ knockout mice	↑	[53]
PPARβ/δ	PPARβ/δ knockdown in cancer cells	↑	[55]
PPARβ/δ	PPARβ/δ agonist GW0742	↑	[29]
PPARβ/δ	Conditional vascular PPARβ/δ Overexpression	↑	[29]
PPARγ	PPARγ agonist rosiglitazone	↓	[58]
PPARγ	PPARγ agonists 15d-PGJ2, rosiglitazone, troglitazone	↓	[59]
PPARγ	PPARγ antagonist T0070907	↓	[50]
PPARγ	PPARγ agonists troglitazone, pioglitazone	↓	[60]
PPARγ	PPARγ agonist RS5444	↓	[61]
PPARγ	PPARγ agonist ciglitazone	↓	[46,62,63]
PPARγ	PPARγ agonist rosiglitazone	↓	[64]
PPARγ	PPARγ agonist rosiglitazone	↓	[65]
PPARγ	PPARγ activation with n-3 fatty acid docosahexaenoic acid (DHA)	↓	[66]
PPARγ	PPARγ agonist rosiglitazone	↓	[67]
PPARγ	Insertion of a constitutively active PPARγ (PγCA) mutant in cancer cells	↑	[68]
PPARγ	PPARγ agonist rosiglitazone	↑	[69]

↑ Indicates an increase, ↓ represents a reduction.

**Table 2:** PPAR modulators in Clinical Studies Related to This Review ^1^.

Identifier	Condition	Intervention	Outcome
NCT00627653 Effect of a PPAR-Alpha Agonist on the Age-Related Changes in Myocardial Metabolism and Mechanical Function	Cardiovascular Diseases	fenofibrate (PPARα agonist)	NC ^2^
NCT00408434 Study of an Experimental New Drug, PPAR Agonist Taken by Mouth by Patients With Advanced or Metastatic Cancer	Neoplasm	CS-7017 (efatutazone, PPARγ agonist)	[163], angiogenesis or effects on vascular cells not investigated
NCT00212004 Pioglitazone Protects Diabetes Mellitus (DM) Patients Against Re-Infarction (PPAR Study)	Diabetes Mellitus Myocardial Infarction	pioglitazone (PPARγ agonist)	NC ^2^
NCT00554853 PPAR-gamma Agonists, Rheumatoid Arthritis and Cardiovascular Disease (RAPPAR)	Cardiovascular Disease Rheumatoid Arthritis	pioglitazone (PPARγ agonist) Sublingual nitroglycerine	[87], vasculoprotective effects
NCT00318617 Effect Of GW501516X On How The Heart Obtains And Uses Energy	Dyslipidemias Heart Failure	GW510516X (PPARβ/δ agonist)	NC ^2^
NCT02152137 A Phase 2 Study of Efatutazone, an Oral PPAR Agonist, In Combination With Paclitaxel in Patients With Advanced Anaplastic Thyroid Cancer	Thyroid Cancer	CS-7017 (efatutazone, PPARγ agonist)	[164], Angiogenesis not investigated
NCT00099021 Pioglitazone Hydrochloride in Preventing Head and Neck Cancer in Patients With Oral Leukoplakia	Head and Neck Cancer	pioglitazone (PPARγ agonist)	71% PR ^3^; 10% SD ^4^; 19% PD ^5^; Angiogenesis not investigated
NCT00003058 Troglitazone in Treating Patients With Liposarcoma	Sarcoma	troglitazone (PPARγ agonist)	[165], Angiogenesis not investigated
NCT00004180 Rosiglitazone in Treating Patients With Liposarcoma	Sarcoma	rosiglitazone (PPARγ agonist)	NC ^2^
NCT00616642 Rosiglitazone in Treating Patients With Pituitary Tumors	Brain and Central Nervous System Tumors	rRosiglitazone (PPARγ agonist)	Terminated due to low patient recruitment
NCT02249949 Efatutazone Dihydrochloride in Treating Patients With Previously Treated Myxoid Liposarcoma That Cannot Be Removed by Surgery	Liposarcoma	efatutazone (PPARγ agonist)	0 out of 11 Complete Response or Partial Response
NCT00552747 Effect of Fenofibrate on Endothelial Function and High-density Lipoproteins (HDL) in Patients With Coronary Heart Disease	Coronary heart disease, hyperlipidemia	fenofibrate (PPARα agonist)	NC ^2^
NCT00322140 CDDO to Treat Solid Tumors and Lymphomas	Solid tumors Lymphoma	CDDO (PPARγ agonist)	NC ^2^
NCT01927315 Effects of Fenofibrate on Endothelial Progenitor Cells in Diabetes	Diabetic Retinopathy	fenofibrate (PPARα agonist)	NC ^2^
NCT03829436 TPST-1120 as Monotherapy and in Combination With (Nivolumab, Docetaxel or Cetuximab) in Subjects With Advanced Cancers	Advanced Cancers	TPST-1120 (PPARα antagonist)	NC ^2^
NCT00115661 Use of Rosiglitazone in the Treatment of Endometriosis	Endometriosis	rosiglitazone (PPARγ agonist)	NC ^2^
NCT03345901 PROMINENT-Eye Ancillary Study (Protocol AD)	Diabetic Retinopathy Diabetic Macular Edema	pemafibrate (PPARα agonist)	NC ^2^
NCT01199068 CS-7017 in Combination With Erlotinib in Subjects With Stage IIIb/IV Non-small Cell Lung Cancer (NSCLC)	Non-Small-Cell Lung Carcinoma	CS-7017 (efatutazone, PPARγ agonist)	NC ^2^
NCT01199055 CS-7017 in Combination With Carboplatin/Paclitaxel in Subjects With Stage IIIb/IV Non-small Cell Lung Cancer (NSCLC)	Non-Small-Cell Lung Carcinoma	CS-7017 (efatutazone, PPARγ agonist)	NC ^2^
NCT02852083 A Trial With Metronomic Low-dose Treosulfan, Pioglitazone and Clarithromycin Versus Standard Treatment in NSCLC (ModuLung)	Squamous Cell Lung Cancer Non-Squamous Cell Lung Cancer Non-Small Cell Lung Cancer	pioglitazone (PPARγ agonist) + nivolumab, treosulfan, clarithromycin	NC ^2^
NCT01504490 Phase I Study of CS-7017 and Bexarotene	Solid Tumors Lymphoma Multiple Myeloma	CS-7017 (efatutazone, PPARγ agonist) and Bexarotene	NC ^2^
NCT00923949 Pioglitazone to Treat Adults Undergoing Surgery for Non-small Cell Lung Cancer	Non-Small-Cell Lung Carcinoma	pioglitazone (PPARγ agonist)	Terminated due to low accrual Angiogenesis not investigated
NCT00951379 Pioglitazone for Oral Premalignant Lesions	Oral Leukoplakia	pioglitazone (PPARγ agonist)	Terminated due to slow accrual 46% response in drug group; 32% response in placebo group Angiogenesis not investigated

^1^ Clinical Studies were identified from the Clinical Trials Database of the National Institute of Health (https://clinicaltrials.gov); ^2^ NC: Not communicated. ^3^ PR: Partial response. ^4^ SD: Stable disease. ^5^ PD: Progressive disease.

## Abbreviations

Akt	Protein kinase B
Angptl4	angiopoietin-like 4
CD36	cluster of differentiation 36
c-Kit	tyrosinkinase KIT
Cox-2	Cyclooxygenase-2
CREB	c-AMP Response Element-binding protein
CRP	C-reactive protein
CXCL	chemokine (C-X-C motif) ligand
EPC	Endothelial progenitor cells
eNOS	Endothelial NO synthetase
Fgf	fibroblast growth factor
GM-CSF	granulocyte-macrophage colony-stimulating factor
11-HETE	11-hydroxyeicosatetraenoic acid
Hif	Hypoxia-inducible factor
HUVEC	Human umbilical vein endothelial cell
IL	Interleukin
K-Ras	Kirsten rat sarcoma viral oncogene homolog
MAPK	Mitogen-activated protein kinase
miR	micro RNA
MSC	mammary stem cell
NC	not communicated
NF-κB	nuclear factor kappa-light-chain-enhancer of activated B cells
NO	Nitric oxide
NOX	NADPH oxidase
PD	Progressive disease
PDGF	platelet-derived growth factor
PDGFR	platelet-derived growth factor receptor
PGC-1α	peroxisome-proliferator-activated receptor-gamma coactivator-1alpha
PGE(2)	prostaglandin E2
PGI2	Prostacyclin
PKC	Protein kinase C
PPAR	Peroxisome proliferator-activated receptor
PPRE	PPAR responsive elements
PR	partial response
Ptn	pleiotrophin
RNA	Ribonucleic acid
SD	Stable disease
SOCS3	suppressor of cytokine signaling 3
TSP-1	Thrombospondin-1
TF	Tissue factor
VEGF	Vascular endothelial growth factor
VEGFR	Vascular endothelial growth factor receptor
